# Inhibition of osteoclastogenesis by RNA interference targeting RANK

**DOI:** 10.1186/1471-2474-13-154

**Published:** 2012-08-22

**Authors:** Ruofan Ma, Jie Xu, Bin Dong, Max Daniel Kauther, Marcus Jäger, Christian Wedemeyer

**Affiliations:** 1 Department of Orthopaedics, Sun Yat-sen Memorial Hospital of Sun Yat-sen University, 107 West Yan Jiang Road, PO Box 510120, Guangzhou, China; 2 Department of Trauma Surgery, University of Duisburg-Essen, Hufelandstr. 55, 45122, Essen, Germany; 3 Department of Orthopaedics, University of Duisburg-Essen, Hufelandstr. 55, 45122, Essen, Germany

**Keywords:** RANK, Inhibition, RNA interference, Cell culture

## Abstract

**Background:**

Osteoclasts and osteoblasts regulate bone resorption and formation to allow bone remodeling and homeostasis. The balance between bone resorption and formation is disturbed by abnormal recruitment of osteoclasts. Osteoclast differentiation is dependent on the receptor activator of nuclear factor NF-kappa B (RANK) ligand (RANKL) as well as the macrophage colony-stimulating factor (M-CSF). The RANKL/RANK system and RANK signaling induce osteoclast formation mediated by various cytokines. The RANK/RANKL pathway has been primarily implicated in metabolic, degenerative and neoplastic bone disorders or osteolysis. The central role of RANK/RANKL interaction in osteoclastogenesis makes RANK an attractive target for potential therapies in treatment of osteolysis. The purpose of this study was to assess the effect of inhibition of RANK expression in mouse bone marrow macrophages on osteoclast differentiation and bone resorption.

**Methods:**

Three pairs of short hairpin RNAs (shRNA) targeting RANK were designed and synthesized. The optimal shRNA was selected among three pairs of shRNAs by RANK expression analyzed by Western blot and Real-time PCR. We investigated suppression of osteoclastogenesis of mouse bone marrow macrophages (BMMs) using the optimal shRNA by targeting RANK.

**Results:**

Among the three shRANKs examined, shRANK-3 significantly suppressed [88.3%] the RANK expression (p < 0.01). shRANK-3 also brought about a marked inhibition of osteoclast formation and bone resorption as demonstrated by tartrate–resistant acid phosphatase (TRAP) staining and osteoclast resorption assay. The results of our study show that retrovirus-mediated shRANK-3 suppresses osteoclast differentiation and osteolysis of BMMs.

**Conclusions:**

These findings suggest that retrovirus-mediated shRNA targeting RANK inhibits osteoclast differentiation and osteolysis. It may appear an attractive target for preventing osteolysis in humans with a potential clinical application.

## Background

Osteoclasts promote bone resorption in metabolic, degenerative and neoplastic bone disorders. Clinically, the mechanisms inhibiting osteoclast formation are potentially important for preventing bone resorption in these bone disorders. Recent studies on cellular and molecular interactions leading to osteolysis have demonstrated a critical role for the receptor activator of nuclear factor 'kappa-light-chain-enhancer' of activated B-cells (NF-κB) and its ligand (RANK/RANKL) [1]. RANKL is expressed on the surface of stromal cells and osteoblasts. It binds to RANK which is expressed on osteoclast progenitors to induce and promote further differentiation into mature osteoclasts [2]. Stromal cells/ osteoblasts also secrete osteoprotegerin (OPG), a soluble glycoprotein of the tumor necrosis factor receptor super-family. OPG acts as a decoy receptor for RANKL, competing against RANK and thereby inhibiting osteoclast differentiation [3]. In addition, the macrophage colony-stimulating factor (M-CSF) is also produced by stromal cells and induces RANKL-like effects in osteoclasts. It is essential for the survival and proliferation of macrophages and the regulation of osteoclastogenesis [4]. Furthermore, various cytokines or hormones influence the complex osteoclast differentiation by regulating expression of RANKL, M-CSF and OPG on stromal cells or osteoblasts [5].

However, the function of RANK is not limited to cell differentiation. As a member of the tumor necrosis factor receptor (TNFR) family RANK is expressed on the surface of osteoclast progenitor cells and plays an important role in bone homeostasis. RANK transduces intracellular signals upon ligand binding by recruiting various adaptor proteins through specific motifs in the cytoplasmic domain [5-11]. Thus, the central role of RANK/RANKL interaction in osteoclastogenesis makes RANK an attractive target for potential therapies.

The aim of this study was to assess the effect of inhibition of RANK expression in mouse bone marrow macrophages (BMMs) on osteoclast differentiation and bone resorption. We also studied the extent of osteoclast inhibition by targeting RANK with retrovirus-mediated shRNA.

## Methods

### Isolation and culture of bone marrow macrophages

All experimental procedures involving animals were performed in compliance with the regulations of the Guide for the Care and Use of Laboratory Animals (NIH publications Nos. 80–23, revised 1996). Furthermore, the procedures complied with the institutional ethical guidelines for animal experiments and approval was received from the institutional review board.

The femora of 5-week-old BALB/c mice fresh cadavers were aseptically removed and dissected free of adhering tissues. The bone ends were cut off with scissors and the marrow cavity was flushed slowly by injecting Dulbecco's Modified Eagle’s Medium (DMEM; Invitrogen, Carlsbad, CA). The bone marrow cells were harvested from one end using a sterile needle. 5 × 10^6^ of these cells were adhered to tissue culture dishes in 10 ml of DMEM containing 10% fetal bovine serum (FBS; Gibico, BRL, Germany), 100 IU/ml penicillin G (Gibico) and 100 μg/ml streptomycin (Gibico) with 100 ng/ml M-CSF (PerproTech, London, UK). Three days later, the floating cells were removed and the attached cells were harvested by treatment with phosphate buffer solution (PBS) and used as bone marrow macrophages (BMMs).

### CD14 and CD34 immunohistochemistry of BMMs

The sections adhered with cells were harvested and washed with PBS, fixed with 95% ethanol for 30 minutes at 25°C, and then washed three times with cold PBS. The sections were placed in a pressure cooker for antigen retrieval using citrate buffer pH6 for 10 minutes. They were then incubated at room temperature and washed with distilled water. After washing, the sections were placed in hydrogen peroxidise 3% for 6 minutes to block endogenous peroxide, washed with water three times and finally with Tris-buffered saline (TBS) for 10 minutes to eliminate non-specific staining. The excess TBS was removed from the slides before incubation with primary antibody.

**Figure 1:**
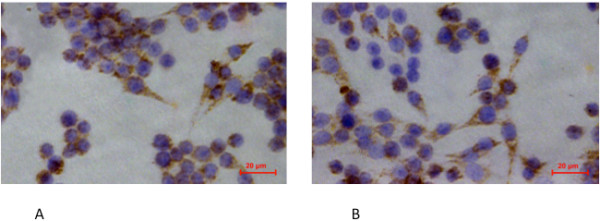
CD14 (A) and CD34 (B) immunohistochemistry of isolated bone marrow macrophages (BMMs).

The immunohistochemical procedure and preparation of controls were carried out according to the manufacturing company’s standards and guidelines. The controls were used to assess the specificity of the reaction as follows: pre-incubation of the cells with the peptide in order to obtain the first antibody, incubation of the cells with an irrelevant primary antibody of the same isotype followed by the second antibody. The sections were incubated with the primary antibodies (CD14 (Santa Cruz, CA, USA, sc-73794) [1:250] and CD34 (Santa Cruz, sc-7045) [1:250]) for 30 minutes at room temperature, washed with TBS and incubated with link antibody for 15 minutes each. Then the sections were washed with TBS and incubated with labeled Streptavidin-biotin (LSAB) for 15 minutes at room temperature, washed again with TBS and incubated with diaminobenzidine (DAB) and substrate chromogen system for 5 minutes at room temperature which resulted in brown-coloured precipitates at the antigen site (Figures 1A-B).

### Cloning into retroviral vectors, selection of the optimal shRNA and stable infection

RANK small hairpin RNA (shRNA) was designed and cloned into the pSUPER-retro-puro retroviral vector (Oligoengine, Seattle, Wash., USA). Three shRNAs were chosen based on the sequence of the mouse RANK gene (GenBank gi: NM_ 009399). They covered different regions of the RANK sequence and showed no homology with non-RANK sequences. A scrambled shRNA was used as a control. The target sequences and corresponding oligonucleotide sequences for the four shRNAs, designated shRANK-1, shRANK-2, shRANK-3 and scramble shRNA, are shown in Table 1. Transfection of cells was carried out with Lipofectamine 2000™ reagent (Invitrogen). Control cells were treated with pSUPER-retro-puro retroviral vector. The relative RANK mRNA and protein levels were determined by Real-time polymerase chain reaction (RT-PCR) and Western blot analyses respectively. The optimal shRANK or vector plasmid and the packaging plasmid PIK were transfected into 293FT cells using the calcium phosphate precipitation method [12,13]. Competent retroviruses were collected 48 hours after transfection. The retroviruses harboring shRANK were transfected into BMMs. Subsequently, the cells were passaged and harvested after treatment in 0.625 μg/ml puromycin (Sigma, St. Louis ,USA) medium for three days. Meanwhile, pSUPER-GFP-retro-puro retroviral vector was used to generate shRANK retrovirus. After transfection, BMMs were passaged and purified by puromycin. The effect of gene silence was also investigated by detection of GFP expression with fluorescence microscopy. 

**Table 1:** Target sequences and corresponding oligonucleotide sequences for the four shRNAs, designated shRANK-1, shRANK-2, shRANK-3 and scramble shRNA

**shRNA**	**Target sequences**	**Oligonucleotide sequences**
shRANK-1	caagaagtgtgtgaaggta	Forward: 5’- -3’ GATCCCCCAAGAAGTGTGTGAAGGTATTCAAGAGATACCTTCACACACTTCTTG TTTTTA
		Reverse:5’- -3’ AGCTTAAAAACAAGAAGTGTGTGAAGGTATCTCTTGAATACCTTCACACACTTCTTG GGG
shRANK-2	tgggcttcttctcagatgt	Forward: 5’- -3’ GATCCCCTGGGCTTCTTCTCAGATGTTTCAAGAGAACATCTGAGAAGAAGCCCA TTTTTA
		Reverse: 5’- -3’ AGCTTAAAAATGGGCTTCTTCTCAGATGTTCTCTTGAAACATCTGAGAAGAAGCCCA GGG
shRANK-3	gcgctgacagctaatttgt	Forward: 5’- -3’ GATCCCCGCGCTGACAGCTAATTTGTTTCAAGAGAACAAATTAGCTGTCAGCGC TTTTTA
		Reverse: 5’- -3’ AGCTTAAAAAGCGCTGACAGCTAATTTGTTCTCTTGAAACAAATTAGCTGTCAGCGC GGG
scrambled shRNA		Forward: 5’- -3’ GATCCCCGCCAGCTTAGCACTGACTCTTCAAGAGAGAGTCAGTGCTAAGCTGGCTTTTT A
		Reverse: 5’- -3’ AGCTTAAAAAGCCAGCTTAGCACTGACTCTCTCTTGAAGAGTCAGTGCTAAGCTGGCGGG

### Isolation of RNA and real time RT-PCR analysis

Total RNA was isolated from cultured cells and purified using the RNeasy Mini Kit (Qiagen, Hilden, Germany). The yield and purity of the RNA was controlled photometrically. RT-PCR was performed in the ABI PRISM® 7000 Sequence Detection System (Applied Biosystems, Foster, USA) using the SYBR Green I master Mix (Applied Biosystems) according to the manufacturer’s instructions. RT-PCR of Glyceraldehyde-3-Phosphate Dehydrogenase (GAPDH), a housekeeping gene, served as a control. Primers used in this study were as follows: GAPDH, 5’- CCAATGTGTCCGTCGTGGAT-3’ (forward) and 5’- TGCTGTTGAAGTCGCAGGAG -3’ (reverse); RANK, 5’ -CTGCCTCTGGGAACGTGACT -3’ (forward) and 5’- GCGAGGTCTGGCTGACATAC-3’ (reverse). Two pairs of primers were added in the action tube for RT-PCR. The PCR cycle conditions were as follows: 2 minutes at 95°C, 30 seconds at 95°C, 35 seconds at 60°C, 40 cycles of 15 seconds at 95°C and 30 seconds at 60°C. Each PCR procedure included a negative control reaction without a template.

### Western blot analysis

The cells were washed three times with ice-cold PBS and lysed in RIPA lysis buffer (Sigma, St. Louis, MO, USA). The lysates with Sodium dodecyl sulphate (SDS) buffer were boiled for 5 minutes and separated by 10% SDS polyacrylamide gel electrophoresis (SDS-PAGE). They were then transferred to a 0.45 μm polyvinylidene difluoride membrane (PVDF; Millipore, Bedford, USA) and incubated with RANK antibodies (Santa Cruz, CA, USA) at a dilution of 1/3000 and Horseradish peroxidase (HRP)-conjugated goat anti- rabbit antibody (Santa Cruz) at a dilution of 1/6000. The HRP substrate was observed on the PVDF membrane. After three washes, the PVDF membrane was incubated with β-actin antibody (Santa Cruz) and HRP-conjugated antibody. The HRP substrate was observed again. For densitometric analyses, blots were scanned and quantified using Quantity One analysis software (Bio-Rad, Hercules, USA), and the results were expressed as the percentages of β-actin immunoreactivity.

### In vitro osteoclastogenesis assays and bone resorption assay

BMMs or retrovirally infected BMMs were cultured in 24-well tissue culture plates (5 × 10^4^ cells/well) in DMEM containing 10% FBS in the presence of 30 ng/ml M-CSF and 100 ng/ml RANKL (PeproTech, London, UK). Osteoclastogenesis began on the sixth day and the cultures were stained for tartrate-resistant acid phosphatase (TRAP) on the ninth day using a kit (Sigma, St. Louis, MO, USA) in accordance with the manufacturer`s instructions. TRAP-positive cells appeared red to purple. Only TRAP-positive cells with more than three nuclei were counted optically. The values were expressed as means and standard deviations of triplicate cultures. Bone resorption assays were performed using osteoclasts generated on mice bone slices from infected or uninfected BMMs as described previously. The bone slices were harvested on the ninth day. Osteoclastic resorption was quantified by measuring the area of resorption pits with digital image analysis software (Image-pro plus 6.0, Media Cybernetics, USA). The percentage of resorption was calculated by dividing the resorbed area by the total area of the imaged bone slice surface.

### Statistical analysis

Data are expressed as mean ± standard deviation. The statistical significance of differences between the experimental and the control groups was evaluated by independent Student’s t test. Differences between the groups of the optimal shRANK selection were analyzed using analysis of variance (ANOVA) with SPSS® 16.0 software (SPSS Inc, Chicago, IL). P <0.05 was considered to be statistically significant.

## Results and discussion

As shown in Figures 1, nearly all cultured BMMs expressed the monocyte/macrophage surface markers CD14 (94.3-0.4%) and CD34 (90.6-3.5%). BMMs also expressed CD11b (94.8-2.9%) and F4/80 (93.1-3.0%), which were the special surface markers of monocyte/macrophage [14]. Flow cytometric analysis on day 7 showed similar patterns of surface marker expression (data not shown).

### RANK mRNA inhibition by RT-PCR

**Figure 2:**
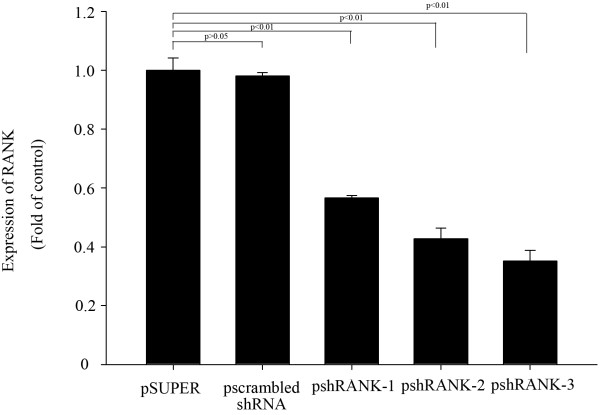
**RANK gene expression inhibited by shRNAs and the inhibition rate of psh RANK-1, psh RANK-2 and psh RANK-3.** Lane 1: pSuper-retro-puro; lane 2: pscrambled shRNA; lane 3: psh RANK-1; lane 4: psh RANK-2; lane 5: psh RANK-3.

The inhibition rates of pshRANK-1, pshRANK-2 and pshRANK-3 were 43.4%, 57.3% and 65.8% (p < 0.01) respectively compared with the control plasmid pSUPER-retro-puro (Figures 2).

### RANK protein inhibition by Western blot

**Figure 3:**
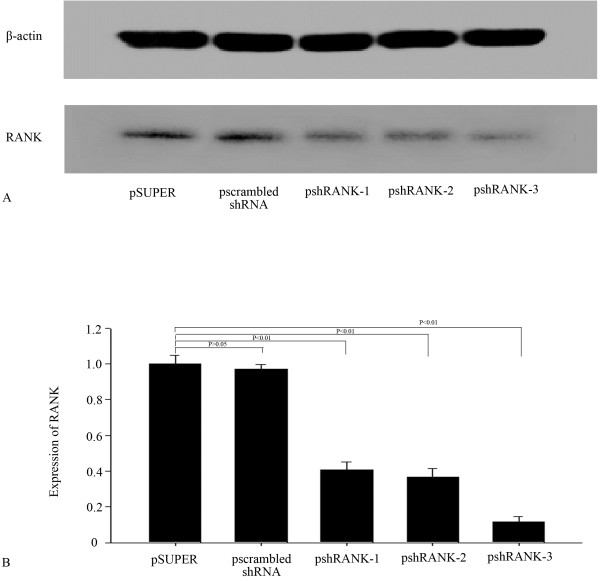
**Western blot for RANK (A) and the inhibition rate of psh RANK-1, psh RANK-2 and psh RANK-3 (B) in BMMs.** Figures 1 RANK gene expression inhibited by shRNAs and the inhibition rate of psh RANK-1, psh RANK-2 and psh RANK-3. Lane 1: Psuper-retro-puro; lane 2: pscrambled shRNA; lane 3: psh RANK-1; lane 4: psh RANK-2; lane 5: psh RANK-3.

The inhibition rates of pshRANK-1, pshRANK-2 and pshRANK-3 were 59.4%, 63.3% and 88.3% (p < 0.01) respectively compared with the control plasmid pSUPER-retro-puro (Figures 3).

### Stable silencing of the RANK in BMMs by retrovirus-mediated shRNA

**Figure 4:**
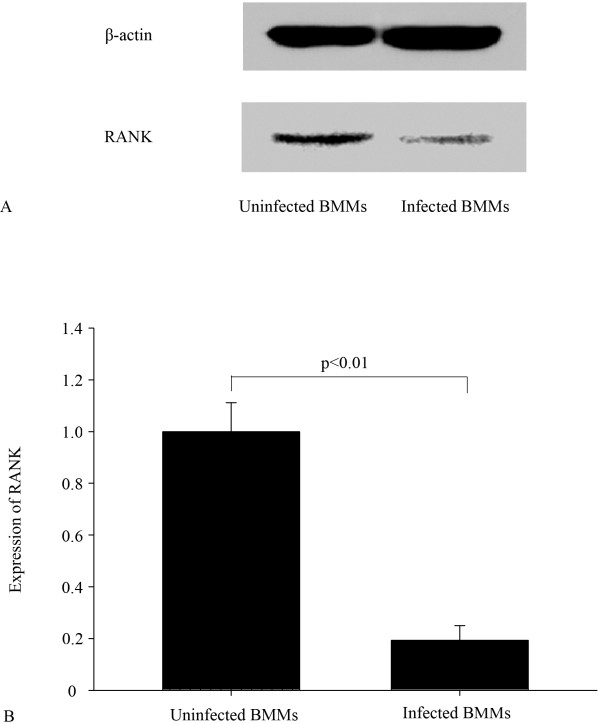
**RANK silencing in BMMs by stable transduction by retroviruses.** BMMs stably transduced with shRNA against the RANK were analyzed for RANK protein levels by Western blot (**A**, **B**); β-actin served as a loading control.

Subsequently, the effect of shRANK-3 on infected BMMs was studied to determine the biological influence of RANK on osteoclastogenesis in BMMs. Western blot was used to evaluate inhibition of RANK expression in stable cells. Expression was reduced by about 80.7% (p < 0.01), as compared with uninfected BMM cells (Figures 4). Meanwhile, the levels of RANK protein were significantly reduced in the 3rd-passage cells, which were used in all experiments in this study. The expression of GFP was continuously monitored with fluorescence microscopy to test whether the cells stably express GFP-retro-puro retrovirus. The 10th-passage cells were detected to stably express GFP protein.

### RANK silencing in BMMs abolishes osteoclast differentiation and osteolysis

**Figure 5:**
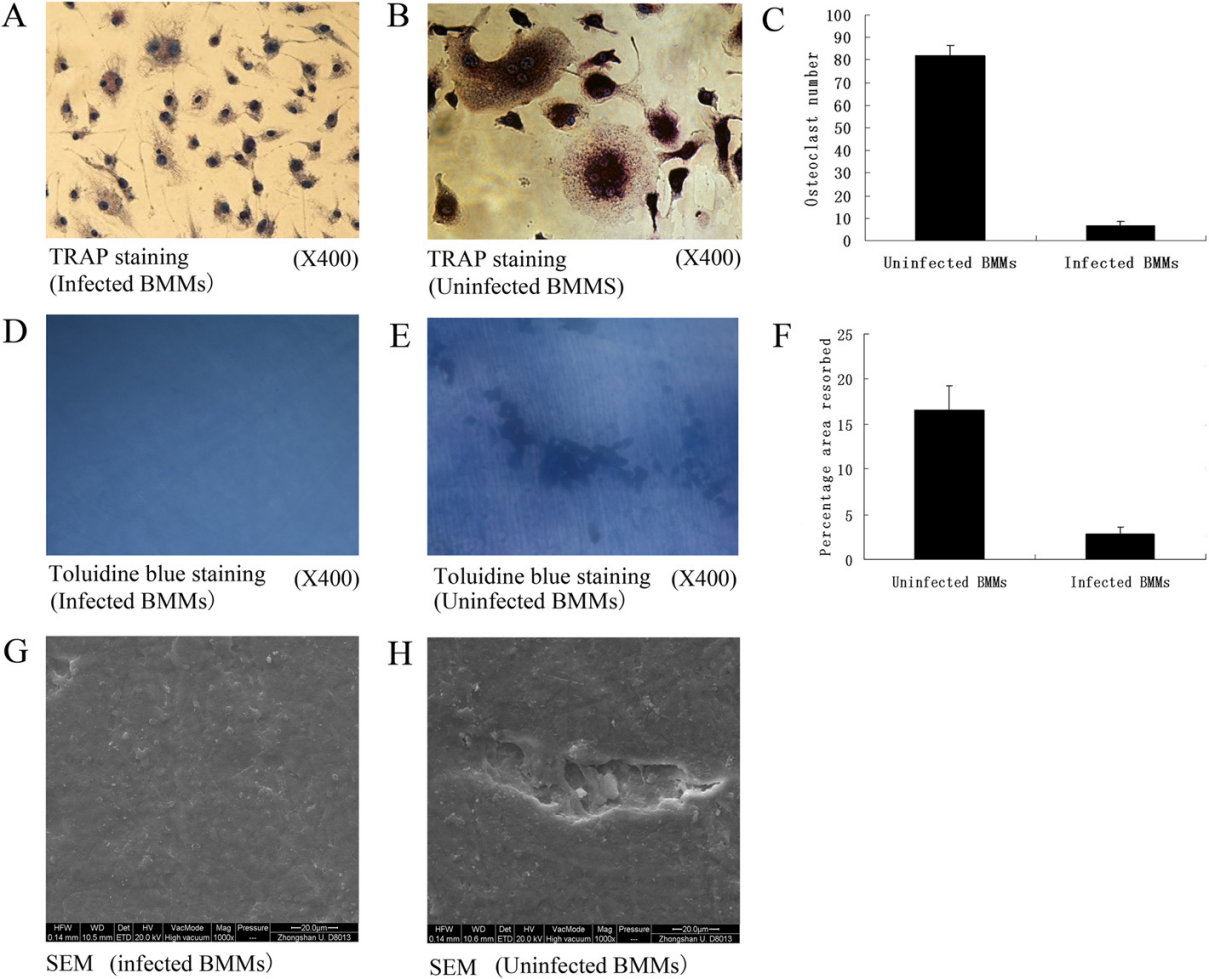
**Inhibition of osteoclastogenesis by RANK silencing.** (**A**, **B**) Osteoclast differentiation of BMMs as identified by TRAP staining. (**C**) Quantification of the data. The multinucleated TRAP-positive cells (>3 nuclei) in eight random view areas (at 200× magnification) in each of the five replicates were counted. Osteoclast resorption was quantified using toluidine blue staining (at 400× magnification) (**D**,**E**,**F**). Comparison of the resorption area of bone slices between untreated and treated BMM cells was evaluated by Scanning Electron Microscopy (at 400× magnification) (**G**, **H**).

We performed functional assays to determine the effect of RANK inhibition on osteoclast differentiation and osteolysis. Infected BMM cells or BMM cells were treated with RANKL (100 ng/ml) and M-CSF (30 ng/ml) for an additional nine days and subjected to TRAP staining and osteoclast resorption assay. TRAP^+^ cells with more than three nuclei were counted and the area of osteoclast resorption was measured with a scanning electron microscope (SEM). We noted a reduction in the number of TRAP^+^ multinucleated cells in infected BMMs (6.8 ± 1.64) compared with BMMs (82 ± 4.64) (p < 0.05) (Figures 5 A-C). Furthermore, a significant reduction in resorption areas on the bone slices (Figures 5 D-F) in infected BMMs (16.6% ± 2.70%) compared with BMMs (2.8% ± 0.84%) (p < 0.05) was observed, indicating profound inhibition of osteoclastic bone resorption. Meanwhile, the resorption area of bone slices between untreated and treated BMM cells was evaluated by Scanning Electron Microscopy (Figures 5 G, H). The difference is obvious.

## Discussion

Osteoclasts (OCs) are multinucleated cells derived from circulating osteoclast precursor cells (OCPs) of the monocyte/macrophage lineage. They represent the only cell type capable of bone resorption [5]. Osteoclasts promote bone resorption in metabolic, degenerative and neoplastic bone disorders. Excess osteoclast activity in osteolysis may involve not only generation and activation of OCs, but also increased recruitment of OCPs. The increased bone resorption in osteolysis is thought to be mediated via numerous pro inflammatory cytokines. These cytokines are considered to act primarily via a common final pathway involving members of the TNF receptor-ligand family: The RANKL and its corresponding RANK receptor that play a crucial role in osteoclast differentiation and activation, and OPG, the physiological inhibitor of RANKL [15]. In turn, excess osteoclast activity is associated with the disorders of the TNF receptor-ligand family that ultimately lead to metabolic, degenerative and neoplastic bone disorders. Clinical treatments are needed that can inhibit excess osteolysis in an inflammatory microenvironment. Given that RANK is the essential signaling receptor for osteoclast differentiation factor in osteoclastogenesis, we tested the hypotheses that inhibition of RANK expression by RNA silencing would reduce the number of osteoclasts and the activity of bone erosion.

The limitation of our study is that BMMs culture experiments have a limited perspective of time as we analyzed the BMM cells for only nine days. As metabolic, degenerative and neoplastic bone disorders are processes which can take years, the reactions by osteoclasts might change in the course of time. The correlational research *in vivo* should be carried on in future studies. Nevertheless, we demonstrated that the difference in osteoclast number and bone erosion between the study groups was significant.

Our data showing inhibition of RANK expression by successful silencing of RNA, which can inhibit specific gene expression, suggests that we can obtain the optimal shRNA-silencing RANK. The inhibition rates of pshRANK-1, pshRANK-2 and pshRANK-3 by Western Blot were 59.4%, 63.3% and 88.3% (p < 0.01) respectively compared with the control plasmid pSUPER-retro-puro, which is consistent with the result of Real-time PCR. Thus, shRANK-3 was identified as the optimal sequence silencing RANK.

Our study shows that inhibition of RANK expression suppresses osteoclast differentiation, thus leading to reduction of bone resorption. We found that RANK gene-silencing using retrovirus-mediated shRNA can significantly suppress RANK expression of BMMs. The inhibition rates of RANK protein by Western Blot was about 80.7% (p < 0.01). TRAP staining and SEM reveal that the osteoclastogenesis of infected BMMs is significantly reduced, compared with uninfected BMMs.

We are not aware of any study showing a similar effect of retrovirus-mediated gene therapy with pshRANK on osteoclastogenesis in BMMs. Although OPG fusion protein and OPG have been used successfully to prevent osteolysis, OPG may bind TNF related apoptosis-inducing ligand in addition to RANKL and thus act as a cancer survival factor [16-18]. Clinical trials with a monoclonal antibody against RANKL showed efficacy and anti-resorptive activity [19], but sensitization to monoclonal antibodies` therapeutics poses significant risk to the patient and may blunt the efficacy of these therapies [20]. In addition, antibodies against OPG fusion protein harbor the potential risk of cross-reacting with and neutralizing endogenous OPG.

Numerous studies have shown that the RANK/RANKL pathway is central to cellular and molecular mechanisms associated with the process of osteolysis [4,15,18].

Subsequently, a gene-silenced RANK gene leads to an imbalance of a variety of downstream signaling pathways which are required for osteoclast development [21]. RANKL binding to RANK induces the trimerization of RANK and TNF receptor-associated factors 6 (TRAF6), which leads to the activation of mitogen-activated kinases (MAPK). TRAF6 adaptor protein binds to the intracellular part of the activated RANK receptor and starts the NF-κB pathway, which finally leads to activation of osteoclastogenesis [22]. Different members of the MAPK family, such as p38-MAPKα and p38-MAPKβ, are known to be involved in osteoclastogenesis [23]. Involvement of p38 mitogen-activated protein kinase signaling pathway in osteoclastogenesis is mediated by receptor activator of NF-κB ligand (RANKL) [23]. Furthermore, an influence on bone metabolism is caused by the induction of the phoshatidylinositol (PI) metabolism, which is mediated by the c-Src kinase. The targeted disruption of the c-Src proto-oncogene leads to osteopetrosis in mice [24]. Following notable advances in the understanding of intracellular signaling pathways of RANK, therapeutic drugs specifically targeting downstream signaling molecules have been developed including Interferon-β, γ, p38 inhibitor (SB203580, FR167653), JNK inhibitor (SB600125), IKappaB kinase (IKK) and NEMO binding domain (NBD peptide) inhibitor, NF-κB inhibitor (NF-κB decoy), Calcineurin inhibitor (Cyclosporin A, FK 506), NFAT inhibitor (VIVIT peptide), PI3K inhibitor (Wortmannin, LY290442) [25-30]. The limitation of these approaches lies in the lack of specificity of these compounds, since these intracellular signaling pathways are also important for the function and homeostasis of other cells and tissues. Furthermore, RANK is expressed by other types of cells (inflammatory cells and endothelial cells) and plays a role in other physiological processes such as inflammation and angiogenesis. This requires the development of a sophisticated cell-specific or tissue-specific drug delivery system to prevent or mitigate their adverse effects [31].

RANK suppression in the RANKL/RANK system appears to be a promising target for potential therapies in the treatment of osteolysis. RNA interference to inhibit specific gene expression has been shown to be an effective and promising technology for both basic science research and therapeutic intervention [32,33].

## Conclusions

Our results suggest that retrovirus-mediated pshRANK suppresses RANK expression of BMMs which further inhibits osteoclastogenesis of BMM treated with M-CSF and RANKL. These findings may provide novel therapeutic strategies for the management of osteolysis. Safety and ethics are areas of concern for employing this therapeutic intervention in humans. In future studies, we would like to investigate the effects of retrovirus-mediated pshRANK on the inhibition of osteoclast differentiation and osteolysis in vivo.

## Abbreviations

NF-kappa B: Nuclear factor `kappa-light-chain-enhancer` of activated B-cells; RANK: Receptor activator of NF- kappa B; RANKL: Receptor activator of NF- kappa B; RNA: Ribonucleic acid; shRNA: Short hairpin ribonucleic acid; MCSF: Macrophage colony-stimulating factor; BMM: Bone marrow macrophages; TRAP: Tatrate-resistant acid phosphatase; OPG: Osteoprotegerin; TNFR: Tumor-necrosis factor receptor; DMEM: Dulbecco`s modified eagle`s medium; PBS: Phosphate buffered solution; TBS: Tris-buffered saline; DAB: Diaminobenzidine; RT-PCR: Realtime-polymerase chain reaction; GAPDH: Glyceralaldehyde-3-phosphate; SDS: Sodium dodecyl sulphate dehydrogenase; HRP: Horseradish peroxidase; ANOVA: Analysis of variance; OC: Osteoclast; OCP: Osteoclast precursor cell; TRAF 6: TNF receptor-associated factor 6; MAPK: Mitogen-activated protein kinase; PI: Phosphatidyl inositol; JNK: c-Jun N-terminal kinase; IKK: IkappaB kinase; NFAT: Nuclear factor of activated T-cells; NBD: NEMO binding domain; PI3K: Inhibitor phosphoinositide 3-kinases.

## Competing interests

This study was financed by the Research Fund of Social Development and the Science and Technology Research of Guangdong Province of China (2009B030801025, 2011B031800156) and by the University of Duisburg-Essen with money given in addition to the DFG (Deutsche Forschungsgemeinschaft) grant of Christian Wedemeyer and Max Daniel Kauther (WE 3634/1-1 and WE 3634/1-2).

## Authors’ contribution

RM and JX carried out the molecular genetic studies and made substantial contributions to the concept and the design of the study, the analysis and the interpretation of the data and drafting of the manuscript. BD carried out the scientific assays and participated in the drafting and critical review of the manuscript. MK participated in the design of the study, performed the statistical analysis and made a critical review of the manuscript. CW conceived the study, participated in its design and coordination and contributed to the drafting of the manuscript. All authors read and approved the final manuscript.

## Pre-publication history

The pre-publication history for this paper can be accessed here:

http://www.biomedcentral.com/1471-2474/13/154/prepub
